# The Housing Bill: Objects and Finance

**Published:** 1919-05-17

**Authors:** 


### THE HOUSING BILL.

### I. Objects and Finance.

This Bill is described in the preamble as "A Bill to amend the Enactments relating to the Housing of the Working Classes, Town Planning, and the Acquisition of Small Dwellings," and, while it purports to amend, it adds another Act to a long series dealing with housing which cover the years 1890 to 1909. The necessity for housing and re-housing has not been suddenly sprung upon the Government. It was before them some years prior to the Great War, and it was known that the last five years, with their total suspension of all building operations, would lead to a much aggravated position.

It therefore seems a great pity, with such ample warning, that the Government did not see their way to bring in an up-to-date consolidating Bill which would sweep away all previous enactments, retaining those parts which have proved useful, expunging useless parts and amending and strengthening existing powers where experience has proved the need. There is a tendency nowadays to hasty and panic legislation, especially where public health is concerned—an epidemic occurs; the Press suddenly discovers that there is overcrowding in cities, causing a heavy death-rate in certain diseases; it occurs to some one else that miners are badly housed and threaten to strike if their conditions are not improved, and immediately a panic occurs among legislators. The result is hurried and ill-considered legislation, the easiest method being legislation "by reference" and the amending of Acts. Both of these methods are the delight of lawyers and the despair of the administrator and laymen, and that the views here expressed are not exaggerations any one can assure himself who takes the trouble to wade through the Housing Acts, 1890-1909. Their difficulties will be increased if they peruse the Bill at present before the House, and it is with the intention of elucidating some of its salient features that this article is penned.

The Bill consists of four parts, which deal respectively with Housing of the Working Classes, Town Planning, Acquisition of Small Dwellings and what are called General Enactments.

### Position of Local Authorities.

The first five sections deal with a very obvious defect in the Housing Act of 1890, viz., the more or less voluntary character of the obligations on the local authority in regard to dealing with the insanitary areas under Parts I and II,, and the making of housing schemes under Part III. If an "official representation" is made under Part I of the Act of 1890, it is stated in Section 3 that "the Local Authority *shall* take such representation into their consideration and, if satisfied of the truth thereof and the sufficiency of their resources, shall pass a resolution to the effect that such is an unhealthy area, etc." There is no occasion to quote further, for, though local authorities have, as a rule, not questioned the truth of the representation, they have frequently refused to act on the plea of insufficiency of their resources. Now this defect has been remedied by the giving of powers to the L.G.B. to replace a defaulting authority by a county council, and, in the event of the latter failing in its duty, to do the work themselves. As regards the making of schemes under Part III of the Act of 1890, a local authority *must* consider the needs of their area and submit a scheme to the L.G.B. within three months of the passing of the new Bill. This is none too peremptory, for delay and procrastination have hitherto dogged the footsteps of most local authorities in dealing with housing. They are further given a time limit in which to carry out the scheme they have formulated. As expense has always been the chief excuse for inaction, it is natural that while drastic powers of compulsion are given for dealing with recalcitrant authorities, no excuse has been left on the score of expense, and this subject is dealt with in Sections 6 to 14.

### Financial Proposals.

In Section 6, the Treasury undertakes to pay the local authority or county council, out of moneys provided by Parliament, such part of the loss as may be determined by the Board with the approval of the Treasury. This means that if the building of houses cannot be made a paying concern as a capital outlay the scheme will be subsidized by the State. Last year the Board advised that they would pay 75% of the loss and the local authority would be responsible for the remaining 25%, but this has been altered and the present proposal is to pay all the deficit which a penny rate will not cover. This proposal has been readily accepted by the Provinces, but, as the *Times* points out in a leading article of their issue of April 30, such an arrangement is unlikely to benefit London at all, as a penny rate in that city produces £180,000, which is likely to make good the loss on any building operations which it is practicable for them to undertake within the next few years. The same article points out that Manchester expects to lose £240,000 annually on its housing scheme when a penny rate only produces £18,000, so that the London rate-payers who pay about one-fifth of the national taxes, would provide Manchester with an annual payment of £44,000 towards its deficit and would not profit themselves by the Government grant. The London County Council, therefore, suggests that as far as London is concerned, the Board should return to their original proposal of paying 75% of the loss. The following section (Section 7) extends the period of repaying loans from thirty to eighty years. Now these arrangements apply to all housing and re-housing schemes and should be a great incentive to local authorities and county councils to undertake schemes at once, and the proviso that the schemes must be carried out within a certain definite period greatly enhances the value of this part.

*(To be continued.)*

